# New Appliances and Things Medical

**Published:** 1909-01-30

**Authors:** 


### NEW APPLIANCES AND THINGS MEDICAL.

[We shall be glad to receive at our Office, 28 & 29 Southampton Street, Strand, London, W.C., from the manufacturers, specimens of new preparations and appliances.]

### SECACORNIN "ROCHE."

(F. Hoffmann, La Roche and Cie., Basel.)

The extensive use of ergot of rye is ample excuse for the labours of chemists and pharmacologists to separate from this complicated substance the various active principles it contains. Recent research has tended to show that the hæmostatic and non-striped muscular stimulant properties of ergot depend upon its alkaloidal constituents. These latter are probably multiple, but to the most important of them the name cornutin or ergotinin has been given. The irritant properties of ergot and its medicinal preparations depend upon the presence of a substance known as sphacelinic acid. In the preparation which we are at present considering, the alkaloids are retained and the irritant principle has been eliminated. Secacornin "roche" is a brown fluid resembling in appearance the official liquid extract of ergot, and may be given by the mouth, or rectum, or by subcutaneous or intra-muscular injection in doses of from 8 to 16 minims.

### SYNTHETIC CAMPHOR.

(E. Schering, Berlin. A. and M. Zimmermann, London.)

A good deal of work has been done recently upon the camphors. By the term camphor the organic chemist understands a definite class of substance—namely, a hydro-aromatic ketone. The best known camphor, however, to the medical man is the white crystalline substance obtained from the camphor laurel (cinnamomum camphora), a native of Formosa and Japan. Recently, however, artificial camphor has been made in the laboratory by means of oxalic acid and turpentine. The natural and artificial products are identical, except in their action on polarised light, the natural being dextrorotatory, the artificial lævorotatory. The therapeutic properties of the different camphors have shown that there is practically no therapeutical difference betwen the natural and artificial product. The camphor before us, made by Messrs. Schering, fulfils the requirements of the British and American Pharmacopœias, and has proved itself therapeutically to be as active as the natural camphor, while the method of production renders it less expensive. We think it is in every sense to be recommended, and will probably find an extensive market.

### IODIPIN TABLETS.

(Merck, Darmstadt.)

For a number of years past the manufacturers of synthetic drugs have been busy in the matter of iodine compounds. The extensive therapeutic use of iodide of potassium and the disagreeable effects produced by this drug in many patients in which its continued use is desirable are certain evidence of the welcome which would be accorded by physicians to any preparation possessed of the medicinal properties of iodide of potassium and not exerting with them the phenomena of so-called iodism. The preparation before us, iodipin, is an organic preparation of iodine possessing the above advantages. It is elegantly dispensed by Messrs. Merck in sugar-coated tablets, each containing 5 grains, an average dose, of the drug. There is ample clinical evidence of the value of iodipin in tertiary syphilis, asthma, scrofula, and rheumatoid arthritis.

